# Supplementary material for: Chromothripsis during telomere crisis is independent of NHEJ, and consistent with a replicative origin
Source: Genome Res. 2019 May;29(5):737–49. doi: 10.1101/gr.240705.118 (PMC6499312; doi:10.1101/gr.240705.118)
Supplement: Supplemental Material [file supp_gr.240705.118_Supplemental_file_1.zip › contigs/annotated_contigs/DB50/contig.2.DB50_length_814_mean_cov_10.3194103194.docx]

**DB50_length_814_mean_cov_10.3194103194**

```
CAATAATTTAGATAGTGAGCTTTTTACATAGATAAGTAGAAAGTTCAATATTTAAAAAGTGACCCCCTTAATTAGGTCCTGCTATAAAT
 >chr5:53280559-53280882 - E=2e-182
GAAGCTGGATTTTACATATGTGAGGGTAAATTGGCAGGATAAACAGCCATAATTGAAGGTCAAAGGAAGTGGCCTAGCTACAGGTTCAG

TCCTTTTCAGTTCTGAATCCTACAAAGATTGCAGGAATCACTTTGAAAATGAAGAAATAAGTGGGGAAGTTGGGCTGAATGTATAAATA

AGGGTTTTCTCAGTAAATTGAAGGCAGAATAATCTAACCTTAGCAAAGAA|AAAAAT|GATTTGCTGCTACTCCCAGATTAAAGAAAGT
                                                   >chr5:53278996-53279493 - E=4e-272
AGACCAATTTACAATTACAGTAGCTATGGTCCAAAATAAAATGCAAATTGTCAAAATGCAACAGTTATAGGGAAACAGGAGTGAACCAG

AGTGCACCTTCTCTGGCATTGTTTTTAGAGAATGGAAGGCTGAAAGTGACATTGCTGAGATAGTGTGATTTAAGCAGCCTTCCCCCACA

TGCTTTGGAGACCAGAGTCCCCAAACTGAAAATTTCAGAAGTTTCTTATAAATGGGTAACATAAATTTGGAGAGCAGATCCAACATTGA

ATGCTGTTTTCCTTTAAGATGCAAGAGGATTAAGTCAATTGATGATTTTTTTAAAGAAAATTTATTTTTAAAATATACTTATGTACAGC

AAAGCACCCTGATAACTCAAAGCAGGTTTACAAGTTTTTCAGGAAAAATCTAGAAAGTGGACAAACAACACTGAATTACCGAATGATTA

GACAGTCTCTATCTT
```
